# Percentage depth dose calculation accuracy of model based algorithms in high energy photon small fields through heterogeneous media and comparison with plastic scintillator dosimetry

**DOI:** 10.1120/jacmp.v17i1.5773

**Published:** 2016-01-08

**Authors:** Ananda Giri Babu Alagar, Ganesh Kadirampatti Mani, Kaviarasu Karunakaran

**Affiliations:** ^1^ Research and Development Centre, Bharathiar University Coimbatore India; ^2^ Department of Radiation Oncology Krishna Institute of Medical Sciences Secunderabad India; ^3^ Department of Radiation Physics Kidwai Memorial institute of Oncology Bangalore India

**Keywords:** TPS algorithms, heterogeneity, percentage depth dose

## Abstract

Small fields smaller than $4\times 4\;{\text{cm}}^{2}$ are used in stereotactic and conformal treatments where heterogeneity is normally present. Since dose calculation accuracy in both small fields and heterogeneity often involves more discrepancy, algorithms used by treatment planning systems (TPS) should be evaluated for achieving better treatment results. This report aims at evaluating accuracy of four model‐based algorithms, X‐ray Voxel Monte Carlo (XVMC) from Monaco, Superposition (SP) from CMS‐Xio, AcurosXB (AXB) and analytical anisotropic algorithm (AAA) from Eclipse are tested against the measurement. Measurements are done using Exradin W1 plastic scintillator in Solid Water phantom with heterogeneities like air, lung, bone, and aluminum, irradiated with 6 and 15 MV photons of square field size ranging from 1 to 4 cm^2^. Each heterogeneity is introduced individually at two different depths from depth‐of‐dose maximum (Dmax), one setup being nearer and another farther from the Dmax. The central axis percentage depth‐dose (CADD) curve for each setup is measured separately and compared with the TPS algorithm calculated for the same setup. The percentage normalized root mean squared deviation (%NRMSD) is calculated, which represents the whole CADD curve's deviation against the measured. It is found that for air and lung heterogeneity, for both 6 and 15 MV, all algorithms show maximum deviation for field size $1\times 1\;{\text{cm}}^{2}$ and gradually reduce when field size increases, except for AAA. For aluminum and bone, all algorithms' deviations are less for 15 MV irrespective of setup. In all heterogeneity setups, $1\times 1\;{\text{cm}}^{2}$ field showed maximum deviation, except in 6 MV bone setup. All algorithms in the study, irrespective of energy and field size, when any heterogeneity is nearer to Dmax, the dose deviation is higher compared to the same heterogeneity far from the Dmax. Also, all algorithms show maximum deviation in lower‐density materials compared to high‐density materials.

PACS numbers: 87.53.Bn, 87.53.kn, 87.56.bd, 87.55.Kd, 87.56.jf

## I. INTRODUCTION

Modern radiotherapy technology dose delivery using intensity‐modulated radiotherapy (IMRT), stereotactic radiosurgery (SRS/SRT), and stereotactic body radiotherapy (SBRT) involves more number of small fields, often less than $4\times 4\;{\text{cm}}^{2}$ in size, to get highly conformal dose distributions and spare nearby critical organs. The target treated also often very much smaller than conventional fractionated radiotherapy and may be present inside or nearer the heterogeneous media with extreme density differences.

The main goal of stereotactic treatments (SRS and SBRT) is to deliver high dose to the target with submillimeter positional accuracy and <3% dose accuracy with steep dose gradient outside the target volume. In the case of simple small field geometries in homogenous medium, the absorbed dose changes rapidly with field size and depth due to the lack of electronic equilibrium.^1^, ^2^ It makes the dosimetry difficult to predict the dose accurately. This is due to the absence of both lateral and longitudinal electronic equilibrium when the field size is smaller than the maximum range of secondary electrons. Introducing the heterogeneous medium inside such small field makes the dose calculation more complex and inaccurate. More variations in tissue density along the path of the beam can produce lot of perturbations depending on the energy of the beam and density variation across the path. When calculating the dose in extreme range of density media such as lung and bone, significant perturbations affect the accuracy of dosimetry. Hence the choice of detector to measure the dose accurately in this complex situation and also the choice of treatment planning systems (TPS) to predict the dose in any combination of medium and energy are important to achieve the results.

The plastic scintillator is one of the suitable detectors for dosimetry of small field with heterogeneity because of its minimal sensitive volume dimensions without air cavity (otherwise, further perturbations are introduced) and water‐equivalent density compared to small volume ionization chambers and diodes.^3^, ^4^, ^5^

The dose prediction accuracy improves only when the TPS uses high standard algorithms where multisource modeling is included to keep track of every secondary scattered photon and electron and its further dose deposition in nonequilibrium conditions. It has been shown that model‐based algorithms significantly improve the dose calculation accuracy when the beam aperture size less than $3\times 3\;{\text{cm}}^{2}$ compared to simple algorithms using one‐ or two‐dimensional density scaling.^(6)^ Hence, four model based algorithms used in clinical treatment planning systems, such as X‐ray Voxel Monte Carlo (XVMC) from Monaco, Elekta, superposition algorithm (SP) from CMS‐Xio, Acuros XB (AcXB), and Analytical Anisotropic algorithm (AAA) from Eclipse, are taken to quantify the accuracy against the respective measured values. Also four heterogeneous materials are studied such as air, lung‐equivalent (Styrofoam fiber slabs), bone‐equivalent (Polyvinylidine fluoride polymer slabs), and aluminum whose respective mass densities are 0.001, 0.27, 1.76, and 2.7 g/cc.

In this study, we are comparing the central axis depth dose(CADD) measured by Exradin‐W1 plastic scintillator in the Solid Water phantom SP‐34 (density = 1.03 g/cc) slabs introduced with one heterogeneous material either nearer or farther from depth‐of‐dose maximum (Dmax) (one heterogeneous material for one study setup), irradiated with one of the two higher energy photons, 6 MV or 15 MV, and opened with one of the field sizes ranging from 1 cm^2^ to 4 cm^2^, with that of same setup dose predicted by each model‐based algorithm mentioned before.

## II. MATERIALS AND METHODS

### A. Measurements

#### A.1 Plastic scintillator

**Figure 1:**
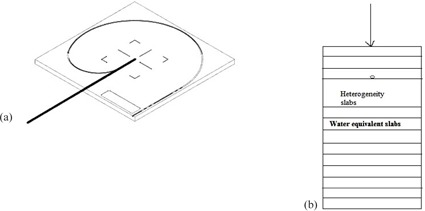
Exradin‐w1 plastic scintillator (a) shown as thick black line in measurement position at the groove and at the center of water‐equivalent slab supplied by Standard Imaging. Another curved groove shown is the position of detector during maximum field calibration. (b) Sample setup showing scintillator plate reversed to measure the dose at entrance junction of heterogenous slab. Detector is shown as small circle and arrow shows the beam entry point on the phantom.

The Exradin‐W1 Plastic scintillator (Standard Imaging, Middleton, WI) is used in which actual sensitive scintillator thickness and length are 1 mm and 3 mm and the same including outer wall are 5 mm and 7 mm. Its minimal sensitive volume (2.3 mm^3^) provide excellent spatial resolution suitable for small field dosimetry.^3^, ^5^ The photons collected due to ionization‐induced scintillation will be transported through the optical fiber and photodiode. At the coupling point between optical fiber and photodiode, the Cherenkov emissions produced is subtracted from actual scintillating photons.^7^, ^8^ This correction must be performed with the help of two optical fiber inbuilt configuration which is part of calibration procedure (to be done with groove carved water‐equivalent slab supplied) (Fig. 1(a)) in using this detector. Final corrected reading gives the absolute or relative values of our choice. The actual calibration and measurement procedure can be studied from the manufacturer manual. Benefits of this detector are well‐documented and include near water equivalence, linear dose and dose rate response, energy independence in MV range above 125 KeV and temperature and directional independence.^(9)^

Before doing the actual measurements in heterogeneity setups, to check the consistency of scintillator detector measurements with small volume 0.13 cc chamber values, the CADD profiles for field sizes 6×6, 8×8, and $10\times 10\;{\text{cm}}^{2}$ are taken both in water and uniform solid water SP‐34 slabs without heterogeneities and found to be within 1% variation.

#### A.2 Measurement setup

The SP34 phantom (IBA Dosimetry AB, Uppsala, Sweden) containing water‐equivalent slabs where heterogeneity slab is introduced in between and the whole phantom of 30 cm thickness including heterogeneity is used. The measurements are done at different depths from surface to bottommost slab including heterogeneity slab of the setup in NovalisTx linear accelerator (Varian Medical Systems, Palo Alto, CA) either with 6 MV or 15 MV photon beam. Depending on the energy in study, each heterogeneity material is kept in two different positions one being nearer and other far from the Dmax of the energy to observe the difference in scatter dose. Table 1 explains the actual study setup for different material and energy configurations.

Scintillator detector can be easily fit into the measurement groove provided at the center of solid water‐equivalent slab supplied along with the detector (Fig. 1(a)). Hence the positional accuracy inside the small field can be achieved accurately. Always groove along with detector facing the beam and it is reversed only at the interface where heterogeneity starts so that the dose at the interface will be accurate (Fig. 1(b)). The readings inside the heterogeneity materials are measured at the holes which are machined to fit the detector exactly, except for air heterogeneity study. For air heterogeneity, the lung‐equivalent slab is cut at the center of slab with area $15\times 15\;{\text{cm}}^{2}$ to make air column and keep the distance between water‐equivalent slabs intact without sag. Once the heterogeneity is over, readings are taken as earlier, till the lower‐most water‐equivalent slab of the setup.

**Table 1:** Measurement study setup showing different heterogeneity material in two different setup for each energy and its position in solid water‐equivalent slab phantom.

*Heterogenous Material / Density*	*Energy of the Beam (MV)*	*Study Setup Near or Far (from Buildup Dmax)*	*Distance from the Beam Entry Surface*	*Material Thickness from Beam Entry Surface*
	6 MV	Near	3 cm	3 cm to 6 cm
Air		Far	7 cm	7 cm to 10 cm
0.001	15 MV	Near	5 cm	5 cm to 8 cm
		Far	10 cm	10 cm to 13 cm
	6 MV	Near	3 cm	3 cm to 6 cm
Lung		Far	7 cm	7 cm to 10 cm
0.27	15 MV	Near	5 cm	5 cm to 8 cm
		Far	10 cm	10 cm to 13 cm
	6 MV	Near	3 cm	3 cm to 6 cm
Bone		Far	7 cm	7 cm to 10 cm
1.76	15 MV	Near	5 cm	5 cm to 8 cm
		Far	10 cm	10 cm to 13 cm
	6 MV	Near	3 cm	3 cm to 8 cm
Aluminum		Far	8 cm	8 cm to 13 cm
2.7	15 MV	Near	5 cm	5 cm to 10 cm
		Far	10 cm	10 cm to 15 cm

Each of the setup defined in the Table 1 is irradiated for four open small square field sizes (1×1, 2×2, 3×3, and $4\times 4\;{\text{cm}}^{2}$) and SSD is kept 100 cm at gantry angle of 0°. Readings are taken in pico coulombs (pc) and converted to relative doses measured at every 5 mm inside the water‐equivalent slab and maximum number of points possible inside the heterogeneous material. More readings are taken near buildup region to fix the Dmax for normalization and at the interfaces. Hence there are four CADD curves (field size 1×1 to $4\times 4\;{\text{cm}}^{2}$) for every study setup in the Table 1, and 16 CADD curves are drawn for every heterogeneity.

### B. Dose Calculation in TPS

As there are four different model‐based algorithms used in the study for comparison. The NovalisTx linear accelerator (Varian Medical Systems) beam modeling parameters required for every algorithm are modeled for the same clinical beam with special attention given to small fields. The same experimental setup mentioned in section A.1 above is replicated while CT scanning and images transferred to respective TPS to get the CADD curves from each algorithm. The dose result type set at dose to water in XVMC and AcuroseXB algorithms even though the dose to medium option available in both. This is done to compare with the dose‐to‐water measurements of water‐equivalent scintillator detector. When dose to water is selected, in nonwater materials this is analogous to calculate the dose received by a volume of water which is small enough to not significantly perturb the energy dependent electron fluence. Due to the very short range of low‐energy electrons, this volume may be much smaller than the dose grid size or the small volume detector.

#### B.1 XVMC algorithm

The XVMC algorithm modeling from Monaco TPS (version 1.6, Elekta AB, Stockholm, Sweden) consists of source modeling, beam collimating system modeling, and patient dose computation. The dose calculation parameters of XVMC for this study are dose calculation grid set at 1 mm, the statistical uncertainty set at 1% per control point, dose result type set at dose to water.

#### B.2 Acuros XB algorithm

The Acuros XB algorithm from Eclipse TPS (version 11, Varian Medical Systems) uses the deterministic radiation transport solutions of the linear Boltzmann transport equation (LBTE) to eliminate the statistical noise in the calculated dose. It directly accounts for the effect of heterogeneities by taking their chemical compositions apart from density.^(10)^ Here the calculation grid size is set at 1 mm and dose result is set at dose to water. Spatial cutoff for photons below 1 KeV and for electron energies below 500 KeV is set inbuilt for patient dose calculation and below which it deposits dose at that voxel itself.

#### B.3 AAA algorithm

The AAA algorithm from (Eclipse TPS version 10, Varian Medical Systems) is used with calculation grid size of 1 mm and heterogeneity correction is applied.

#### B.4 Superposition algorithm

The standard multigrid superposition algorithm from CMS‐Xio (TPS Version 4.8, Elekta AB) is used which has more accuracy than fast superposition. Here also grid size is kept at 1 mm and inbuilt heterogeneity correction is applied.

### C. Comparison and calculations

**Figure 2:**
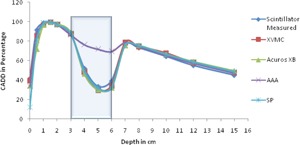
CADD curves of measured and four algorithms for lung heterogeneity in 6 MV near to Dmax setup for field size $1\times 1\;{\text{cm}}^{2}$. Lung heterogeneity region is shown in shaded area.

All the dose calculation parameters like calculation volume, normalization, and grid size are kept same in all the algorithms. For each algorithm, as mentioned earlier, 16 CADD curves are generated for every heterogeneity and compared with the respective measured curve. To compare all algorithm‐generated CADD curves of an energy (either 6 or 15 MV) and a field size (one among the square field size from 1 to 4 cm^2^) calculated on a setup (heterogeneity either far or near to buildup) against the respective measured CADD curve, it is plotted in a single graph and compared. Hence each graph contains five CADD curves, four from each algorithm against one from measured. For example, Fig. 2 represents CADD curves of a measured and four algorithms for lung heterogeneity in 6 MV near to Dmax setup for field size $1\times 1\;{\text{cm}}^{2}$. As there are 64 graphs in total, only four graphs (2, 3, 4, 5) are shown as examples.

To measure the deviation of each algorithm calculated point in the curve against the measured one and to represent the deviation of full curve of an algorithm with a single value, the percentage normalized root mean squared deviation (%NRMSD) is calculated. This represents the root mean square deviation (RMSD) of an algorithm's CADD curve points against the measured one and normalized with respect to measured maximum to minimum CADD difference. The formula is

(1) $$RMSD=\sqrt{1/n\sum_{i=1}^{n}{\left(Measured_{i}-\text{Alg}\;calculated_{i}\right)}^{2}}$$

where *n* is the number of depth points for which CADD is calculated. The percentage NRMSD is calculated as

(2) $$\%NRMSD=\frac{RMSD}{\left(Measured_{\text{max}}-Measured_{\text{min}}\right)}\times 100$$

**Figure 3:**
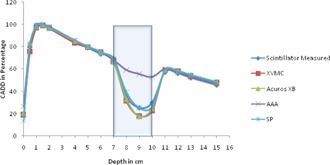
CADD curves of measured and four algorithms for lung heterogeneity in 6 MV far to Dmax setup for field size $1\times 1\;{\text{cm}}^{2}$. Lung heterogeneity region is shown in shaded area.

**Figure 4:**
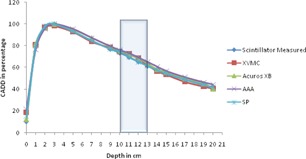
CADD curves of measured and four algorithms for bone heterogeneity in 15 MV far to Dmax setup for field size $4\times 4\;{\text{cm}}^{2}$. Bone heterogeneity region is shown in shaded area.

**Figure 5:**
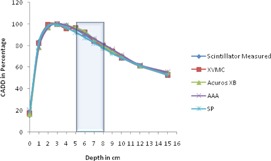
CADD curves of measured and four algorithms for bone heterogeneity in 15 MV near to Dmax setup for field size $4\times 4\;{\text{cm}}^{2}$. Bone heterogeneity region is shown in shaded area.

## III. RESULTS

The graphical results of curve between field size and %NRMSD for every energy and setup are shown in 6, 13.

**Figure 6:**
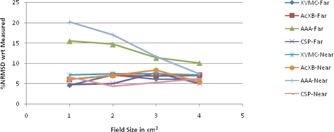
Curves of %NRMSD of calculated CADD curves with respect to measured for every field size in two different air heterogeneity setups for 6 MV photon beam.

**Figure 7:**
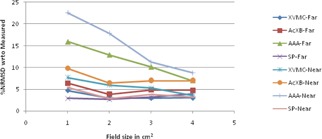
Curves of %NRMSD of calculated CADD curves with respect to measured for every field size in two different air heterogeneity setups for 15 MV photon beam.

**Figure 8:**
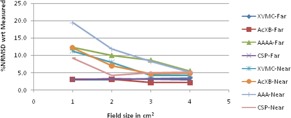
Curves of %NRMSD of calculated CADD curves with respect to measured for every field size in two different lung heterogeneity setups for 6 MV photon beam.

**Figure 9:**
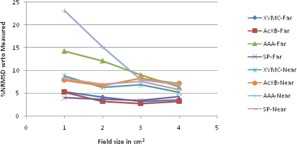
Curves of %NRMSD of calculated CADD curves with respect to measured for every field size in two different lung heterogeneity setups for 15 MV photon beam.

**Figure 10:**
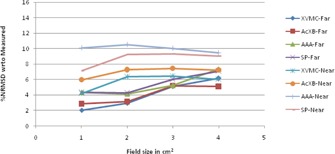
Curves of %NRMSD of calculated CADD curves with respect to measured for every field size in two different bone heterogeneity setups for 6 MV photon beam.

**Figure 11:**
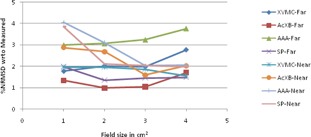
Curves of %NRMSD of calculated CADD curves with respect to measured for every field size in two different bone heterogeneity setups for 15 MV photon beam.

**Figure 12:**
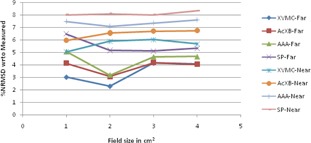
Curves of %NRMSD of calculated CADD curves with respect to measured for very field size in two different aluminum heterogeneity setups for 6 MV photon beam.

**Figure 13:**
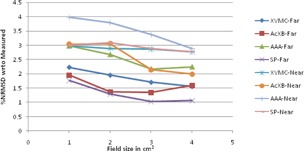
Curves of %NRMSD of calculated CADD curves with respect to measured for every field size in two different aluminum heterogeneity setups for 15 MV photon beam.

### A. Air heterogeneity

For 6 MV photons in air heterogeneity (Fig. 6), the %NRMS deviation from measured for all algorithms are between 4.2 to 8.28%, except AAA whose deviation for $1\times 1\;{\text{cm}}^{2}$ is as high as 20.1% for near setup and 15.5% for far setup. However, as field size increases, this deviation for AAA is gradually becoming less and at $4\times 4\;{\text{cm}}^{2}$ it is the same as other algorithms. Also XVMC in near Dmax setup and Acuros XB in far setup show deviation around 7% irrespective of field sizes.

The same pattern followed for 15MV (Fig. 7.) also. The %NRMS deviation from measured for all algorithms are between 2.7 to 7% except AAA whose deviation for $1\times 1\;{\text{cm}}^{2}$ is high as 22.6% for near setup and 15.96% for far setup. Even when field size is increasing, the deviation for AAA is reducing but higher than the other algorithms. For both the energies and setups, SP has the least deviation of all, between 4% to 7%.

### B. Lung heterogeneity

For 6 MV lung heterogeneity far setup (Fig. 8), the %NRMS deviation is between 2.2% to 3.5% for all algorithms, except for AAA where deviation for $1\times 1\;{\text{cm}}^{2}$ is 12.3%, and gradually reducing to 5.5% for $4\times 4\;{\text{cm}}^{2}$. However in near setup, the deviation for all algorithms is above 9.2% and gradually reducing around 4.3% to 5.1% for $4\times 4\;{\text{cm}}^{2}$. For 6 MV lung setup, Acuros XB had the least variation in far setup and SP algorithm in near setup.

Even though for 15 MV (Fig. 9) the same pattern is followed, the percentage deviation is 2% to 3% higher than the 6 MV values. The %NRMS deviation is between 3.2% and 8.8% for all algorithms, except for AAA where deviation for $1\times 1\;{\text{cm}}^{2}$ is 14.2% (far setup) and 23% (near setup) and gradually reducing to 6.4% (far setup) and 6.8% (near setup) for $4\times 4\;{\text{cm}}^{2}$. For 15 MV lung setups, Acuros XB had the least variation in far setup and XVMC had the least in near setup.

### C. Bone heterogeneity

For 6 MV bone heterogeneity far setup (Fig. 10), the %NRMS deviation is 2.3% to 4.3% for $1\times 1\;{\text{cm}}^{2}$ and increases gradually to maximum deviations (5% to 7.3%) for $4\times 4\;{\text{cm}}^{2}$ in all algorithms. In near setup, all the deviations are higher than far setup and deviations increase from $1\times 1\;{\text{cm}}^{2}$ (4.2% to 10.1%) to $2\times 2\;{\text{cm}}^{2}$ (6.3% to 10.5%) and again decreased for $4\times 4\;{\text{cm}}^{2}$ (6% to 9.5%). For bone 6 MV, in both setups, XVMC showing the least deviation of all.

For 15 MV bone heterogeneity far setup (Fig. 11), the deviation is less (1.0% to 2.7%) for all field sizes in all the algorithms, except AAA where deviations are 3.0% to 3.76% and Acuros XB shows least deviation in all field sizes. Whereas in near setup, inverse to 6 MV near setup, deviations maximum at $1\times 1\;{\text{cm}}^{2}$ and decreasing up to $3\times 3\;{\text{cm}}^{2}$ and again little increase in deviation values at $4\times 4\;{\text{cm}}^{2}$. However XVMC shows very little variations in deviations irrespective of field size, with maximum overall deviation being 2.76%.

### D. Aluminum heterogeneity

For 6 MV aluminum heterogeneity far setup (Fig. 12), the deviation is in the range of 2.3% to 6.5% for all field sizes of all algorithms where SP showing maximum deviations and least with XVMC. In near setup, the deviations observed in the range of 5.1% to 8.3% for all algorithms, having SP and AAA maximum deviation and least with XVMC algorithm.

For 15 MV aluminum heterogeneity far setup (Fig. 13), the deviation is in the range of 1% to 3.0% for all field sizes and algorithms. The maximum deviation observed for $1\times 1\;{\text{cm}}^{2}$ in all algorithms. In near setup, the deviations are in the range of 2.0% to 4.0% where maximum deviations observed with AAA Algorithm.

## IV. DISCUSSION

This study compared CADD calculation accuracy of four model based algorithms against the measured data in small fields with heterogeneities irradiated by 6 and 15 MV photons. But the previously reported data^11^, ^12^, ^13^ compared some or all the algorithms mentioned in this study with theoretical Monte Carlo data only. Hence this study is unique in comparing the algorithm calculated data with the measured using plastic scintillator, taking advantage of special features of this detector as mentioned earlier. Also most of the reported articles compared only for 6 MV and the heterogeneity at one position in the homogenous water‐equivalent media.

As the deviation measured in this study is NRMSD with respect to measured for the full CADD curve, the variation obtained may be on little higher side than the previous conventional Monte Carlo comparison studies. Generally in all the setups of this study, more deviations from the measured are observed at the interfaces and inside the heterogeneities. In air heterogeneity for 6 MV and 15 MV and in lung heterogeneity for 15 MV deviations are between 3% to 8% irrespective of near or far setups for all the algorithms except AAA which shows highest deviation of 23% for near and 16% for far setup. Higher deviations are found in $1\times 1\;{\text{cm}}^{2}$ fields, which may be due to lateral and forward scatter of secondary particles are not modeled accurately in AAA compared to other algorithms. But only in lung heterogeneity for 6 MV near setup even XVMC, AcXB, and SP also show higher deviations between 9% and 13% for $1\times 1\;{\text{cm}}^{2}$ field, same as AAA. In 6 MV bone and aluminum setups, SP and AAA algorithms show more deviation of 7% to 10%, whereas XVMC and AcXB show in the range of 2% to 7% for all the field sizes. In 15 MV bone and aluminum the least deviation of the whole study is found within 3% for all the algorithms and deviation of 4% observed only for $1\times 1\;{\text{cm}}^{2}$ in AAA and near setup of SP in bone. In high‐density material setups, all the algorithms model the secondary scatter dose deposit accurately (comparatively better than lower density setups) due to fewer scatter components and show less deviation.

The results presented here may involve statistical uncertainties in the calculations of commercial algorithms and the same may reflect in the calculation of NRMS deviation. Also it is difficult to report the random error present in the measurements. Hence multi‐institutional studies of same sort should be conducted to evaluate the accuracy of respective algorithms in small fields with heterogeneities, as more of SBRT, SRS, and IMRT treatments are becoming standard clinical practice.

### V. CONCLUSIONS

All algorithms in the study, irrespective of energy and field size, when any heterogeneity is nearer to depth‐of‐dose maximum, the absolute dose deviation with respect to measured is higher compared to the same heterogeneity far from the Dmax. Also all algorithms shows maximum deviation in lower density materials like air and lung compared to high‐density materials like bone and aluminum. This may be due to the reduced accuracy of algorithms in modeling the increased lateral scatter phenomena in low‐density materials than in high‐density materials. For 15 MV, in high‐density material setups all the algorithms shows less than 4% absolute dose deviation and for 6 MV; in air setups, all algorithms shows more than 4.3% absolute dose deviation. Except in bone heterogeneity, for other heterogeneity setups, $1\times 1\;{\text{cm}}^{2}$ field sizes show maximum deviation due to the maximum range of electrons than the field dimension. In low‐density material setups, for both energies AAA show highest RMSD; however, as the field size increases, it decreases and becoming equal with other algorithms. In high‐density material near setups, both AAA and SP algorithms show larger deviation than XVMC and Acuros XB. Hence dose evaluation in stereotactic treatments of lung and spine using model‐based algorithms, target nearer to Dmax should be done carefully, especially for the field size smaller than $2\times 2\;{\text{cm}}^{2}$.

## ACKNOWLEDGMENTS

We would like to thank all the staff in the department of Radiation Oncology, Krishna Institute of Medical Sciences for all their support in this work and Mr. James Mazerello from Rosalina Instruments for providing plastic scintillator and measuring systems.

## Supporting information

Supplementary Material

Supplementary Material

Supplementary Material

Supplementary Material

Supplementary Material

Supplementary Material

Supplementary Material
