# Supplementary figures and images for: Percentage depth dose calculation accuracy of model based algorithms in high energy photon small fields through heterogeneous media and comparison with plastic scintillator dosimetry
Source: J Appl Clin Med Phys. 2016 Jan 8;17(1):132–42. doi: 10.1120/jacmp.v17i1.5773 (PMC5690200; doi:10.1120/jacmp.v17i1.5773)

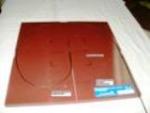

Supplement: Supplementary file 1 — Supplementary Material [file ACM2-17-132-s001.JPG]

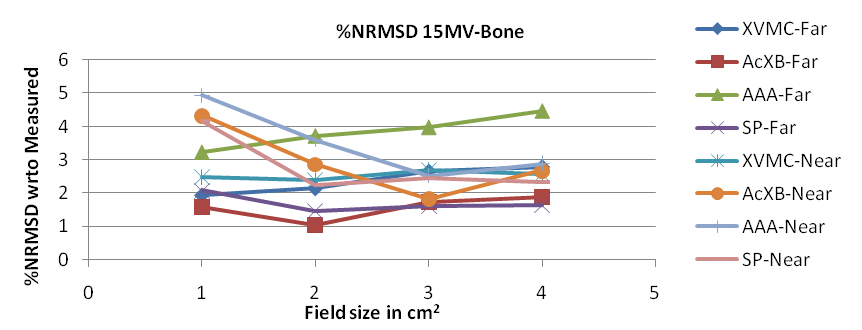

Supplement: Supplementary file 2 — Supplementary Material [file ACM2-17-132-s002.png]

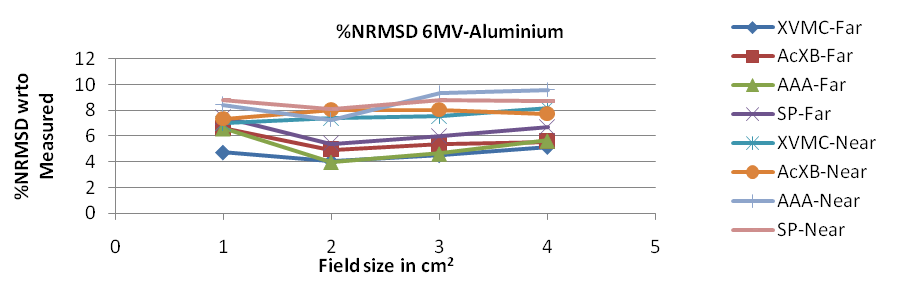

Supplement: Supplementary file 3 — Supplementary Material [file ACM2-17-132-s003.png]

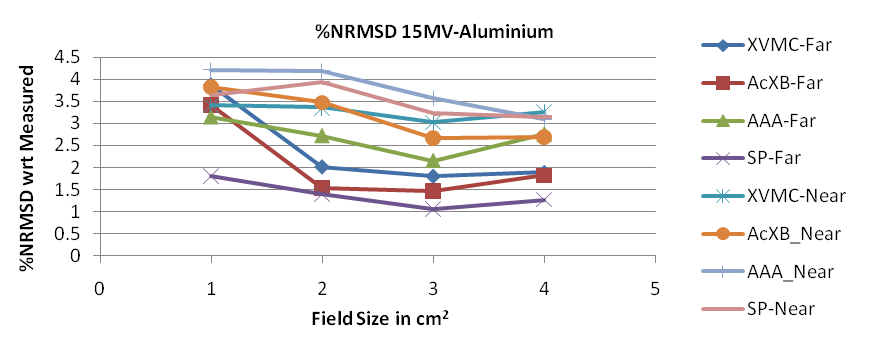

Supplement: Supplementary file 4 — Supplementary Material [file ACM2-17-132-s004.png]

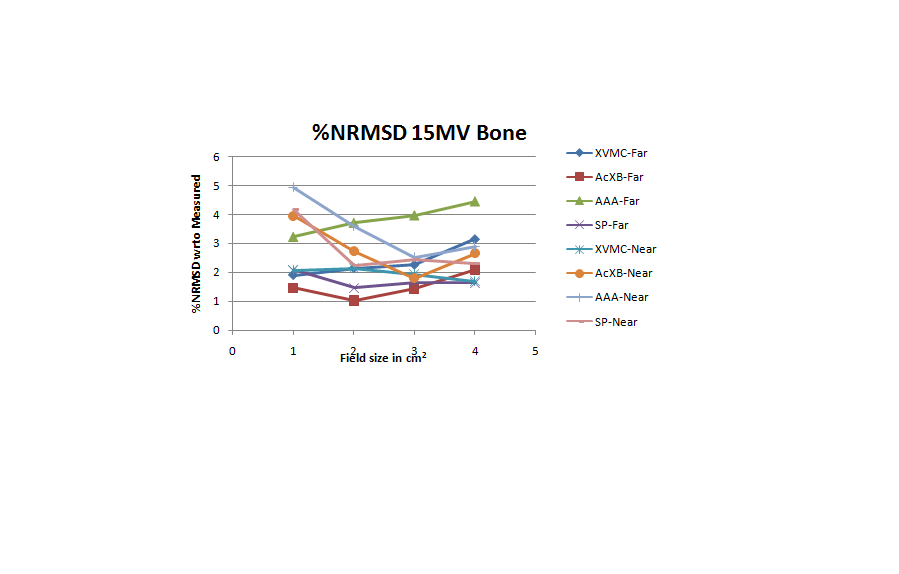

Supplement: Supplementary file 5 — Supplementary Material [file ACM2-17-132-s005.png]

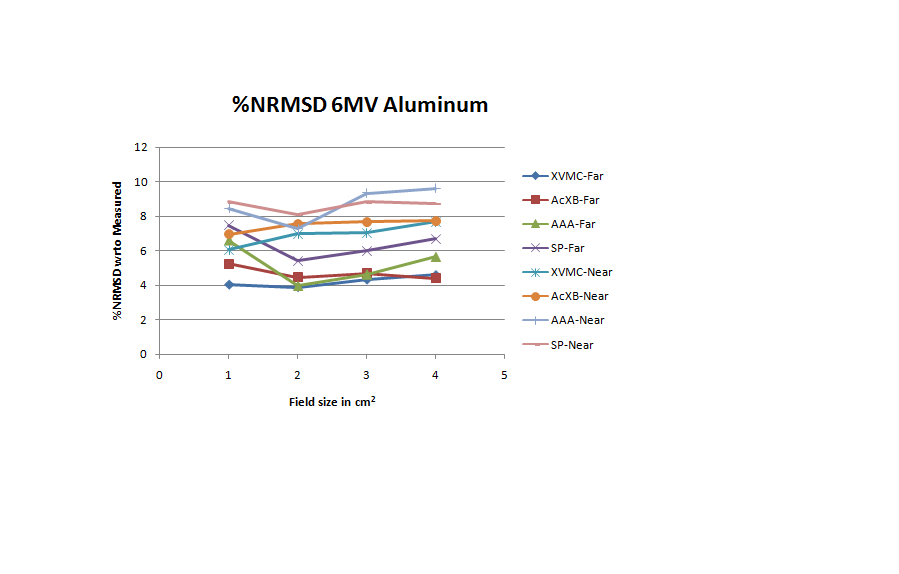

Supplement: Supplementary file 6 — Supplementary Material [file ACM2-17-132-s006.png]

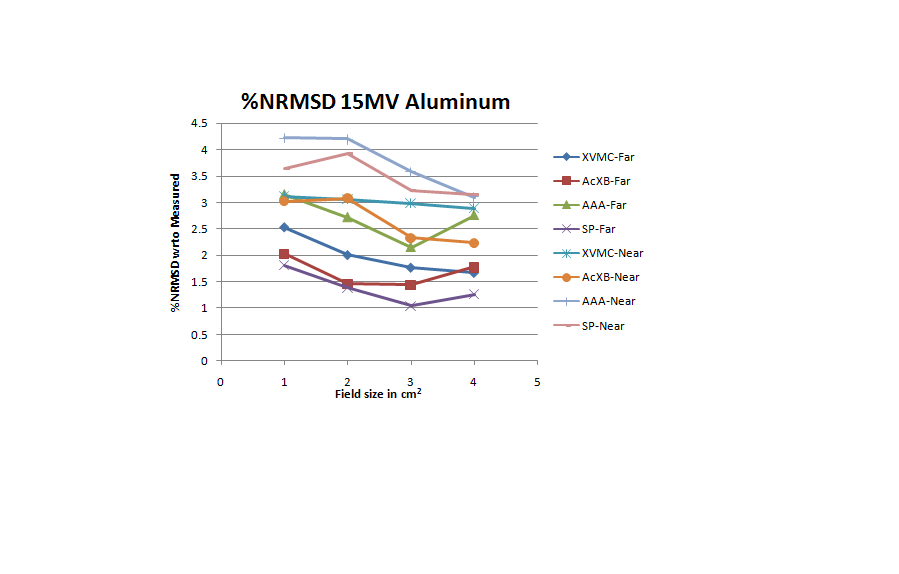

Supplement: Supplementary file 7 — Supplementary Material [file ACM2-17-132-s007.png]
